# Identification of Plasma Biomarkers for B7 Family Members Associated With Primary Sjögren's Syndrome

**DOI:** 10.1002/iid3.70250

**Published:** 2025-08-22

**Authors:** Saizhe Song, Yanhong Yang, Yu Shen, Tian Ren, Zhiyong Sun, Cuiping Liu

**Affiliations:** ^1^ Jiangsu Institute of Clinical Immunology The First Affiliated Hospital of Soochow University Suzhou China; ^2^ Department of Obstetrics and Gynecology Affiliated Hospital of Nantong University Nantong China; ^3^ Department of Rheumatology The First Affiliated Hospital of Soochow University Suzhou China; ^4^ Department of Orthopedics Kezilesu Kirgiz Autonomous Prefecture People's Hospital Xinjiang China; ^5^ Department of Orthopedics The First Affiliated Hospital of Soochow University Suzhou China

**Keywords:** biomarker, disease activity, plasma protein, primary Sjögren's syndrome

## Abstract

**Objectives:**

To evaluate the plasma levels of B7 family members (B7‐H1, B7‐H2, B7‐H3, B7‐H4, B7‐H5, and B7‐H6) in primary Sjögren's syndrome (pSS) patients and investigate their potential associations with disease activity.

**Methods:**

This study included 69 pSS patients and 59 healthy participants. The expression levels of six costimulatory molecules were measured using enzyme‐linked immunosorbent assay (ELISA). Furthermore, we examined the potential correlations between the levels of soluble B7‐H1 (sB7‐H1), sB7‐H2, sB7‐H3, sB7‐H4, sB7‐H5, and sB7‐H6 with clinical symptoms and laboratory parameters.

**Results:**

pSS patients showed significantly higher expression levels of sB7‐H1, sB7‐H2, and sB7‐H5 compared to healthy controls (HCs) (*p* < 0.05). In contrast, sB7‐H6 expression levels were significantly lower in pSS patients (*p* < 0.05). Correlation analysis revealed that sB7‐H1 exhibited positive associations with RF, IgG, CRP, and ESR. sB7‐H2 showed significant positive correlations with both IgG and ESR, while sB7‐H3 exhibited a negative correlation with RF and a positive correlation with CRP. Additionally, sB7‐H5 revealed significant positive correlations with RF, IgG, and ESR. In contrast, sB7‐H6 demonstrated negative correlations with IgG, IgA, and ESR. ESSDAI‐related analysis revealed a significant positive correlation between sB7‐H1 and ESSDAI (*p* < 0.05), while sB7‐H6 exhibited a significant negative correlation with ESSDAI (*p* < 0.05). sB7‐H1 exhibited an increasing trend in patients with clinical symptoms such as xerostomia, xerophthalmia, decayed tooth, fatigue, arthralgia, and glandular swelling, as well as in those with high IgG levels and positivity for anti‐SSB/La, anti‐SSA/Ro60, and anti‐SSA/Ro52. Conversely, sB7‐H6 demonstrated a declining trend in these patient groups. The combined use of sB7‐H1 and sB7‐H6 demonstrates good effectiveness in diagnosing pSS and distinguishing disease activity levels.

**Conclusion:**

Our results indicated that patients with pSS exhibited elevated expression of sB7‐H1 and a reduction in sB7‐H6. These changes were found to correlate with clinical symptoms and laboratory parameters, suggesting that sB7‐H1 and sB7‐H6 could potentially serve as biomarkers for pSS.

## 1 Introduction

Primary Sjögren's syndrome (pSS) is a chronic autoimmune disease characterized by lymphocytic infiltration of exocrine glands, notably the lacrimal and salivary glands, resulting in sicca symptoms [1]. The assessment of pSS typically incorporates immunologic, histopathologic, ophthalmologic, and salivary functional parameters. Conventional biomarkers for pSS comprise indirect immunofluorescence assay on HEp‐2 cells (HEp‐2 IFA), anti‐Sjögren's syndrome‐related antigen A (SS‐A/Ro), rheumatoid factor (RF), and anti‐Sjögren's syndrome‐related antigen B (SS‐B/La). Currently, there are no established diagnostic criteria for pSS, only classification criteria are available. As a result, some patients may still be misdiagnosed with pSS, highlighting the need for further research to identify novel diagnostic biomarkers [2].

PSS is a systemic autoimmune condition marked by aberrant T cell‐driven B cell activation and elevated secretion of pro‐inflammatory cytokines [3]. The B7 family plays a pivotal role in modulating immune activity by maintaining immune homeostasis. Encompassing both co‐stimulatory and co‐inhibitory ligands, it serves as a fundamental pathway in the regulation of T‐cell activation [4]. PD‐L1, also referred to as B7‐H1 [5], exerts its immunoregulatory effects by engaging with PD‐1, thereby functioning as a critical immune checkpoint that suppresses T cell activation [6]. The engagement of B7‐H2 with CD28 plays a crucial role in delivering costimulatory signals that initiate naïve T cell responses to allogeneic antigens and support subsequent memory T cell activation [7]. Initially characterized as a costimulatory molecule enhancing the expansion of CD4⁺ and CD8⁺ T lymphocytes, B7‐H3 was subsequently found to exert suppressive functions by attenuating T cell activation, proliferation, and cytokine secretion [8]. B7‐H4 functions as an immune checkpoint modulator, influencing multiple biological processes such as T cell activation, cytokine secretion, and the regulation of tumor growth and invasiveness [9]. B7‐H5 exerts immunosuppressive effects on T cells by attenuating their activation, limiting proliferative capacity, and reducing cytokine production [10]. B7‐H6, as a natural ligand of NKp30, initiates NK cell‐mediated cytotoxic responses [10]. Notably, NK cells contribute significantly to the immunopathogenesis of pSS [11].

Nonetheless, the potential of co‐stimulatory molecules such as sB7‐H1, sB7‐H2, sB7‐H3, sB7‐H4, sB7‐H5, and sB7‐H6 as biomarkers for pSS has not been thoroughly explored. Thus, our goal is to evaluate the expression of these six soluble costimulatory molecules in the plasma of pSS patients and analyze their clinical correlations.

## 2 Materials and Methods

### 2.1 Patients and Healthy Controls

Sixty‐nine patients with pSS who satisfied the 2016 American College of Rheumatology (ACR)—European League Against Rheumatism (EULAR) classification criteria for pSS [12, 13] were consecutively enrolled from April 2022 to October 2022 through the Department of Rheumatology and Immunology at the first affiliated Hospital of Soochow University, Suzhou, China. Recruited patients were diagnosed for the first time. Patients exhibiting Sjögren's features in the context of other established connective tissue diseases (CTDs), including systemic lupus erythematosus (SLE)and rheumatoid arthritis (RA), were excluded from the study. Additionally, individuals with conditions associated with xerophthalmia and/or xerostomia, such as sarcoidosis, hepatitis B or C, acquired immunodeficiency, history of head and neck radiation, pre‐existing lymphoma, or graft‐versus‐host disease, were also excluded. Healthy controls (HCs) were recruited from the Health Examination Center of the First Affiliated Hospital of Soochow University. For eligible participants, patient‐specific variables were systematically documented. Plasma samples were collected via centrifugation and stored at −80°C. The clinical characteristics of the patients are summarized in Table 1. In addition, the study was approved by the Ethics Review Board of the First Affiliated Hospital of Soochow University (Ethical no. 2021087), and informed consent was obtained.

**Table 1:** Basic information of enrolled study subjects.

Characteristic	Patients with pSS (*n* = 69)	Healthy controls (*n* = 59)
Demographic		
Female, *n* (%)	67 (97.10%)	55 (93.22%)
Age, mean (SD), years	48.86 ± 11.31	49.45 ± 10.32
Disease duration mean (SD), months	24 (10,60)	NA
Clinical manifestation, *n* (%)		
Xerostomia *n* (%)	58 (84.06%)	NA
Xerophthalmia (%)	42 (60.87%)	NA
Decayed tooth, *n* (%)	24 (34.78%)	NA
Glandular swelling, *n* (%)	8 (11.59%)	NA
Raynaud's phenomenon, *n* (%)	6 (8.70%)	NA
Fatigue, *n* (%)	22 (31.88%)	NA
Weight loss, *n* (%)	3 (4.35%)	NA
Arthralgia, *n* (%)	16 (23.19%)	NA
Cutaneous manifestations, *n* (%)	3 (4.35%)	NA
Major laboratory features		
ESR (mm/h)	20 (7, 36)	NA
CRP (mg/L)	1.47 (0.72, 2.66)	NA
RF (IU/mL)	77.65 (28.20, 129.60)	NA
WBC (×10^9^/L)	5.08 (3.46, 6.17)	NA
Lymphocyte (×10^9^/L)	1.41 (1.10, 1.99)	NA
NC (×10^9^/L)	2.75 (2.09, 3.72)	NA
Hb (g/L)	123 (113, 132)	NA
Plt (×10^9^/L)	181.70 ± 65.43	NA
IgG (g/L)	16.70 (13.45, 21.60)	NA
IgA (g/L)	2.99 (2.06, 4.17)	NA
IgM (g/L)	1.36 (0.77, 1.87)	NA
C3 (g/L)	0.83 (0.77, 0.92)	NA
C4 (g/L)	0.18 (0.14, 0.22)	NA
Anti‐Ro52 (+), *n* (%)	56 (81.16%)	NA
Anti‐Ro60 (+), *n* (%)	50 (72.46%)	NA
Anti‐SSB (+), *n* (%)	32 (46.38%)	NA
Anti‐His (+), *n* (%)	3 (4.35%)	NA
Anti‐centromere (+), *n* (%)	8 (11.59)	NA
ESSDAI	4 (2, 8)	NA

Abbreviations: C3, complement 3; C4, complement 4; CRP, C‐reactive protein; ESR, erythrocyte sedimentation rate; ESSDAI, European League Against Rheumatism Sjogren's Syndrome Disease Activity Index; Hb, hemoglobin; IgA, immunoglobulin A; IgG, immunoglobulin G; IgM, immunoglobulin M; NA, not applicable; Nc, neutrophil cell; PLT, platelet; RBC, red blood cell; RF, rheumatoid factor; WBC, white blood cell.

### 2.2 Enzyme‐Linked Immunosorbent Assay (ELISA)

After thawing plasma samples, the assay was conducted following the manufacturer's instructions for reagent preparation, sample addition, incubation, washing, color development, and colorimetric measurement. Concentrations were calculated based on the standard curve. The following commercially available ELISA kits were used for detection: sB7‐H1 ELISA kit (XG‐K3002, Bright Scistar Biotech, Suzhou, China), sB7‐H2 ELISA kit (XG‐K3003, Bright Scistar Biotech, Suzhou, China), sB7‐H3 ELISA kit (XG‐K3004, Bright Scistar Biotech, Suzhou, China), sB7‐H4 ELISA kit (XG‐K3008, Bright Scistar Biotech, Suzhou, China), sB7‐H5 ELISA kit (XG‐K3014, Bright Scistar Biotech, Suzhou, China), and sB7‐H6 ELISA kit (XG‐K3018, Bright Scistar Biotech, Suzhou, China).

### 2.3 Statistical Analysis

Statistical analysis was conducted using GraphPad Prism version 8.0 or SPSS version 25.0 software. Data are presented as means ± standard error of the mean (SEM). For normally distributed data, the *t*‐test was employed to determine significance, while the Mann–Whitney *U*‐test was applied for non‐normally distributed data. Pearson's correlation coefficient was used for correlation analysis of normally distributed data, and the Spearman correlation coefficient was used otherwise. Additionally, the diagnostic performance of plasma proteins was assessed using the area under the receiver operating characteristic (ROC) curve (AUC). Disease activity was stratified based on ESSDAI scores, with values < 5 indicating low activity and ≥ 5 representing moderate to high activity [14]. This classification served as the basis for disease activity differentiation and the construction of related ROC curves. A *p* value of < 0.05 was considered statistically significant. (*p < 0.05, **p < 0.01, ***p < 0.001, ****p < 0.0001).

## 3 Results

### 3.1 Clinical Characteristics and Laboratory Parameters of Included Participants

Table 1 provides a comparative analysis of the clinical characteristics between patients diagnosed with pSS and HCs. Notably, no significant differences were observed in terms of gender distribution or age between the two groups (*p* > 0.05).

### 3.2 Elevated Expression of sB7‐H1, sB7‐H2, and sB7‐H5, and Decreased Expression of sB7‐H6 in the Plasma of pSS Patients

**Figure 1:**
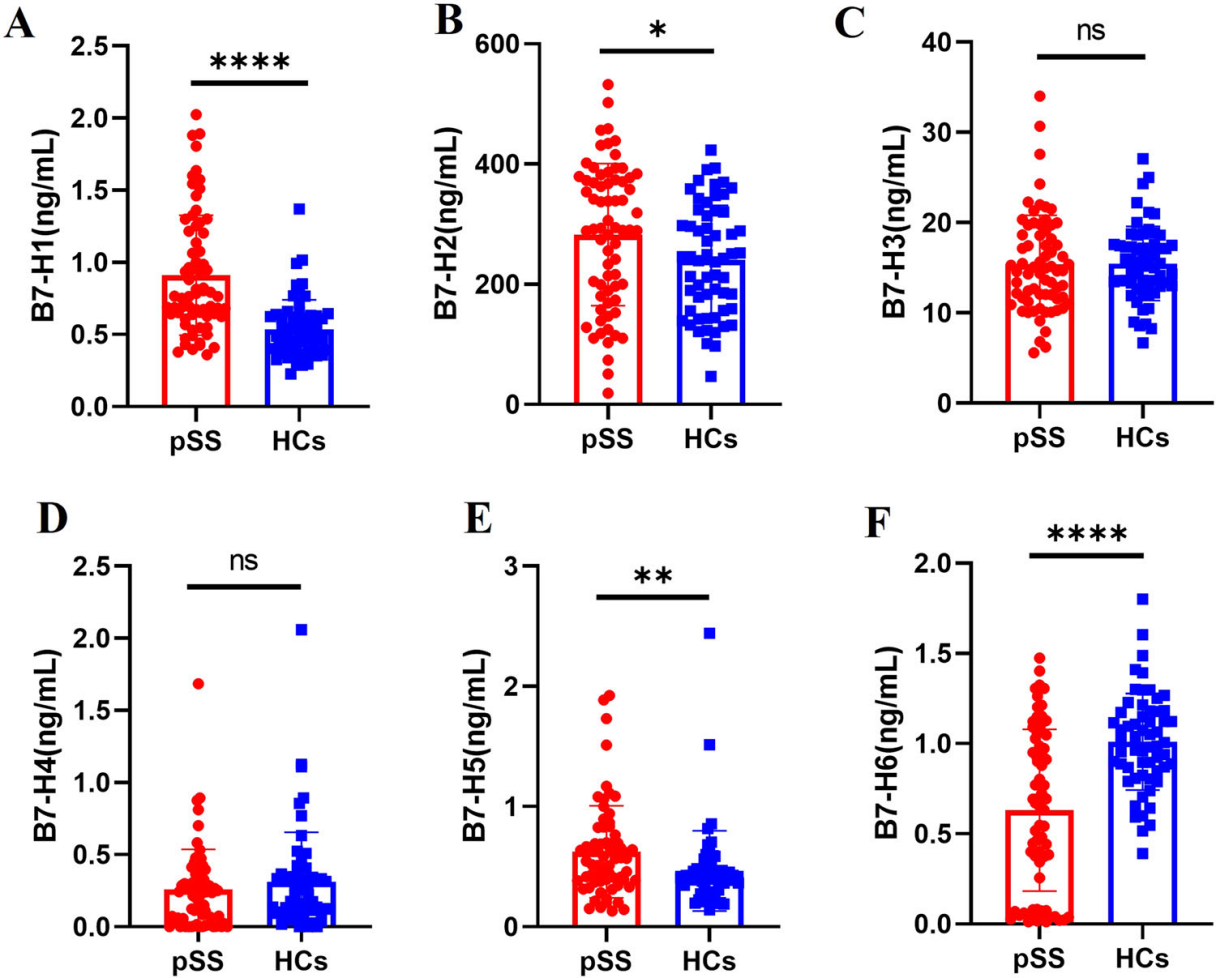
The expression levels of sB7‐H1, sB7‐H2, sB7‐H3, sB7‐H4, sB7‐H5 and sB7‐H6. The expression levels of sB7‐H1(A, *p* < 0.0001), sB7‐H2 (B, *p* = 0.03), sB7‐H5 (E, *p* = 0.002) were higher in patients with pSS compared to HCs. There were no significant statistical differences observed in the expression of sB7‐H3 (C, *p* = 0.58) and sB7‐H4 (D, *p* = 0.30) between patients with pSS and HCs. The expression levels of sB7‐H6 (F, *p* < 0.0001) were lower in patients with pSS compared to HCs. Data of Figure 1 A, C, D, E and F used the Mann–Whitney *U*‐test for statistical difference analysis, **p < 0.01; ****p < 0.0001; ns: not significant (*p* >0.05) Data of Figure 1B used *t*‐test for statistical difference analysis, *p < 0.05.

Plasma concentrations of sB7‐H1, sB7‐H2, sB7‐H3, sB7‐H4, sB7‐H5, and sB7‐H6 were measured using ELISA. The results showed that sB7‐H1 (*p* < 0.0001), sB7‐H2 (*p* = 0.03), and sB7‐H5 (*p *= 0.002) were significantly elevated in pSS patients compared to HCs (Figure 1A,B,E). In contrast, sB7‐H6 levels were notably reduced in pSS patients (*p* < 0.0001), while sB7‐H3 (*p* = 0.58) and sB7‐H4 (*p *= 0.30) showed no significant differences between the two groups (Figure 1C,D,F).

### 3.3 Correlation Between Plasma Proteins and Clinical Characteristics in pSS Patients

Additional investigations were performed to examine the associations between sB7‐H1, sB7‐H2, sB7‐H3, sB7‐H4, sB7‐H5, sB7‐H6, and clinical assessments (Table 2). sB7‐H1 were positively correlated with RF, IgG, CRP, and ESR (RF: *r* = 0.37, *p* = 0.03; IgG: *r* = 0.31, *p* = 0.01; CRP: *r* = 0.27, *p* = 0.04; ESR: *r* = 0.40, *p* = 0.001). sB7‐H2 were positively correlated with IgG and ESR (IgG: *r* = 0.27, *p* = 0.03; ESR: *r* = 0.26, *p* = 0.04). sB7‐H3 were negatively correlated with RF (*r* = −0.36, *p* = 0.03). Conversely, sB7‐H3 were positively correlated with CRP (*r* = 0.25, *p* = 0.04). Moreover, sB7‐H5 were positively correlated with RF, IgG, and ESR (RF: *r* = 0.35, *p* = 0.04; IgG: *r* = 0.29, *p* = 0.02; ESR: *r* = 0.28, *p* = 0.03). sB7‐H6 were negatively correlated with IgG, IgA, and ESR (IgG: *r* = −0.32, *p* = 0.009; IgA: *r* = −0.43, *p* = 0.0004; ESR: *r* = −0.38, *p* = 0.004).

**Table 2:** Correlation between six plasma proteins and clinical parameters in pSS patients.

	B7‐H1		B7‐H2		B7‐H3		B7‐H4		B7‐H5		B7‐H6	
	*r* value	*p* value	*r* value	*p* value	*r* value	*p* value	*r* value	*p* value	*r* value	*p* value	*r* value	*p* value
RF	0.37	0.03*	0.19	0.23	−0.36	0.03*	0.10	0.58	0.35	0.04*	−0.19	0.26
IgG	0.31	0.01*	0.27	0.03*	−0.10	0.45	0.09	0.50	0.29	0.02*	−0.32	0.009**
IgA	0.01	0.93	0.18	0.15	−0.04	0.74	−0.20	0.11	0.03	0.80	−0.43	0.0004***
IgM	−0.07	0.55	0.12	0.35	−0.01	0.93	−0.13	0.30	−0.04	0.73	−0.06	0.62
C3	−0.14	0.27	−0.06	0.64	0.02	0.85	0.10	0.41	−0.03	0.82	0.10	0.50
C4	−0.14	0.27	0.04	0.75	0.16	0.21	−0.07	0.60	−0.08	0.50	0.01	0.94
CRP	0.27	0.04*	0.05	0.69	0.25	0.04*	−0.07	0.61	−0.06	0.65	−0.18	0.17
ESR	0.40	0.001**	0.26	0.04*	0.03	0.82	−0.15	0.23	0.28	0.03*	−0.38	0.004**

*Note:* Spearman correlation analysis was performed, *p < 0.05; **p < 0.01; ***p < 0.001.

### 3.4 Association Between Six Plasma Proteins and ESSDAI Scores in pSS Patients

**Figure 2:**
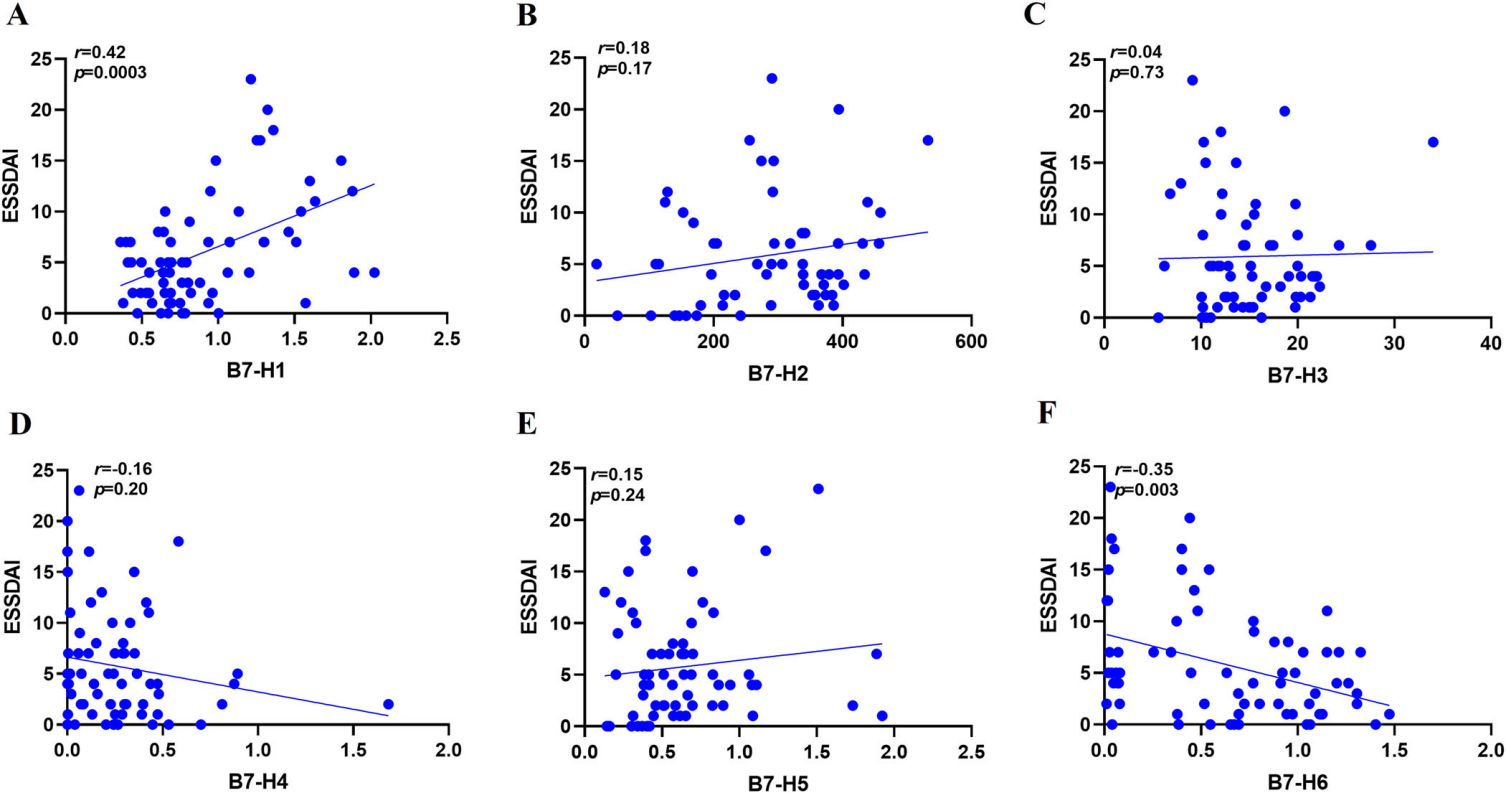
Correlation of the expression levels of sB7‐H1, sB7‐H2, sB7‐H3, sB7‐H4, sB7‐H5 and sB7‐H6 with ESSDAI scores in pSS. (A) The expression of sB7‐H1 in pSS patients was positively correlated with ESSDAI (*r* = 0.42, *p* = 0.0003). (B) sB7‐H2 and ESSDAI scores show a weak positive correlation, and the association is not statistically significant (*r* = 0.18, *p* = 0.17). (C) sB7‐H3 and ESSDAI scores show an extremely weak positive correlation, and the association is not statistically significant (*r* = 0.04, *p* = 0.73). (D) sB7‐H4 and ESSDAI scores show a weak negative correlation, and the association is not statistically significant (*r* = −0.16, *p* = 0.20). (E) sB7‐H5 and ESSDAI scores show a weak positive correlation, and the association is not statistically significant (*r* = 0.15, *p* = 0.24). (F) The expression of sB7‐H6 in pSS patients was negatively correlated with ESSDAI (*r* = −0.35, *p* = 0.003). All of the above correlation analyses were conducted using Spearman's rank correlation.

To further explore the clinical significance, the correlation between the six plasma proteins and ESSDAI scores in pSS patients was assessed. Notably, the plasma concentration of sB7‐H1 showed a positive correlation with ESSDAI (*r* = 0.42, *p *= 0.0003) (Figure 2A), while sB7‐H6 exhibited a negative correlation with ESSDAI (*r* = −0.35, *p *= 0.003) (Figure 2F). sB7‐H2 showed a weak positive correlation with ESSDAI (*r* = 0.18, *p *= 0.17) (Figure 2B), while sB7‐H3 had an extremely weak positive correlation (*r* = 0.04, *p *= 0.73) (Figure 2C). sB7‐H4 exhibited a weak negative correlation (*r* = −0.16, *p *= 0.20) (Figure 2D), and sB7‐H5 showed a weak positive correlation (*r* = 0.15, *p *= 0.24) (Figure 2E), but none were statistically significant.

### 3.5 Expression Levels of sB7‐H1 and sB7‐H6 Across Different Clinical Features in pSS Patients

**Figure 3:**
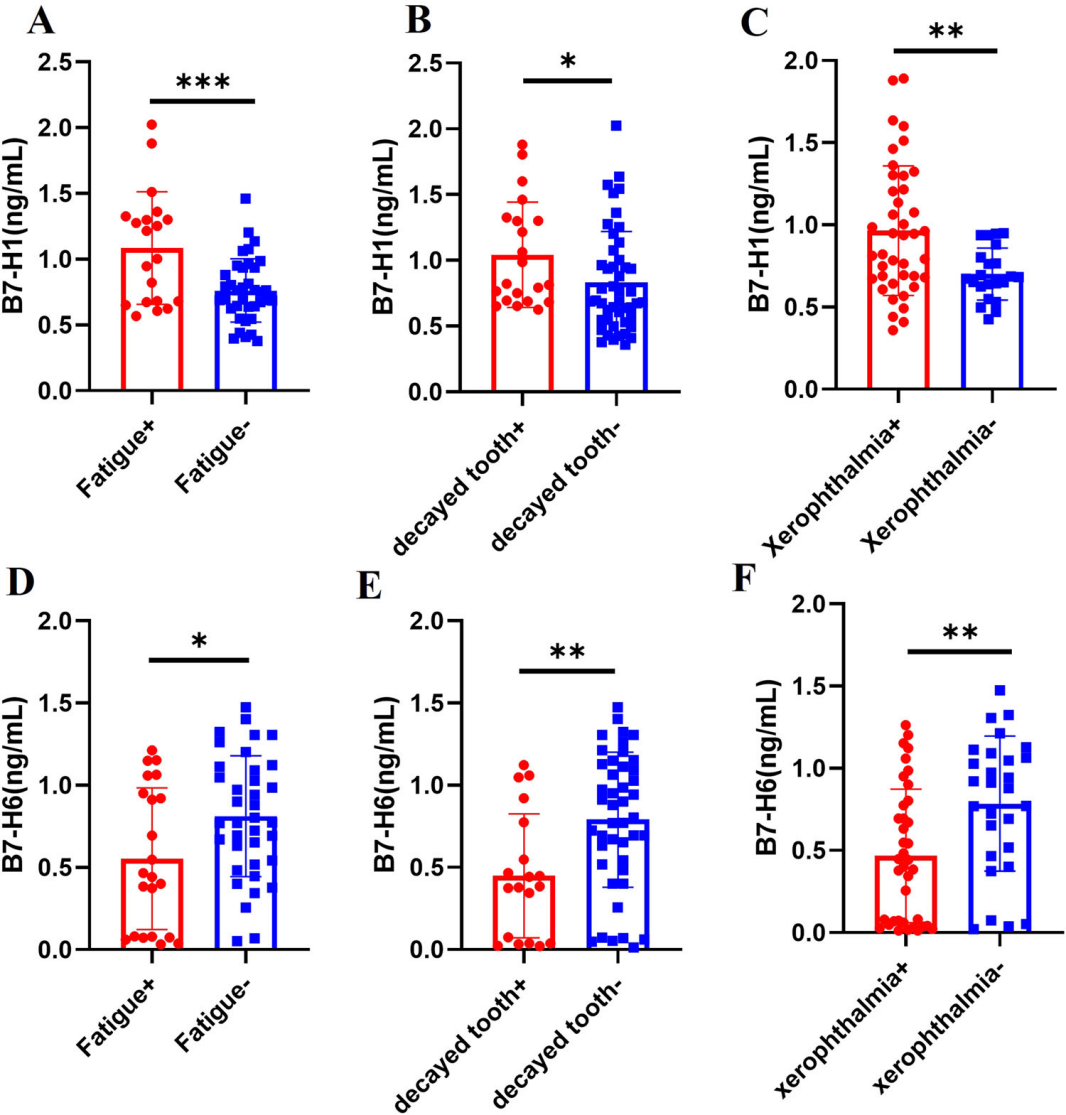
The expression levels of sB7‐H1 and sB7‐H6 in the different clinical features of pSS patients. sB7‐H1 increased markedly in patients with fatigue (A, *p* = 0.0006), decayed tooth (B, *p* = 0.02), xerophthalmia (C, *p* = 0.004). sB7‐H6 decreased markedly in patients with fatigue (D, *p* = 0.04), decayed tooth (E, *p* = 0.002), xerophthalmia (F, *p* = 0.004). Data of Figure 3 B, D, E and F used the Mann– Whitney *U*‐test for statistical difference analysis, *p < 0.05; **p < 0.01. Data of Figure 3 A and C used *t*‐test for statistical difference analysis, **p < 0.01; ***p < 0.001.

We aimed to assess whether changes in the expression levels of sB7‐H1 and sB7‐H6 were associated with clinical features such as fatigue, decayed tooth, xerophthalmia, xerostomia, arthralgia, and glandular swelling. The results indicated that sB7‐H1 expression was significantly elevated in patients with fatigue ((1.09 ± 0.43) vs. (0.76 ± 0.24), *p *= 0.0006) (Figure 3A), decayed tooth(0.82(0.69,1.31) vs. 0.69(0.55,1.00), *p *= 0.02) (Figure 3B), and xerophthalmia((0.97 ± 0.39) vs. (0.70 ± 0.16), *p *= 0.004) (Figure 3C). Conversely, sB7‐H6 expression was significantly reduced in patients with fatigue (0.45(0.08, 0.98) vs. 0.77(0.53, 1.10), *p *= 0.04) (Figure 3D), decayed tooth (0.38(0.04, 0.77) vs. 0.84(0.52, 1.12), *p *= 0.002) (Figure 3E) and xerophthalmia (0.42(0.06, 0.78) vs. 0.90(0.48, 1.08), *p *= 0.004) (Figure 3F).

We also evaluated changes in the expression of sB7‐H1 and sB7‐H6 in pSS patients with xerostomia, arthralgia, and glandular swelling. The results showed that sB7‐H1 levels were significantly increased in patients with xerostomia (0.82(0.67, 1.31) vs. 0.64(0.43, 0.69), *p *= 0.0002) (Figure S1A). Although sB7‐H1 was elevated in patients with arthralgia (0.87(0.64, 1.50) vs. 0.77(0.62, 1.04), *p *= 0.51) (Figure S1B) and glandular swelling (1.22(0.64, 1.33) vs. 0.76(0.63, 1.09), *p *= 0.50) (Figure S1C), these differences were not statistically significant. Conversely, sB7‐H6 levels were significantly decreased in patients with xerostomia (0.54(0.07, 0.99) vs. 0.91(0.70, 1.06), *p *= 0.02) (Figure S1D) and glandular swelling (0.37(0.01, 0.47) vs. 0.70(0.08, 1.06), *p *= 0.04) (Figure S1F). Although sB7‐H6 was also reduced in patients with arthralgia (0.46(0.12, 0.75) vs. 0.72(0.08, 1.06), *p *= 0.19) (Figure S1E), the difference was not statistically significant.

### 3.6 The Expression Levels of sB7‐H1 and sB7‐H6 in Autoantibody‐Positive and High IgG Levels pSS Patients

**Figure 4:**
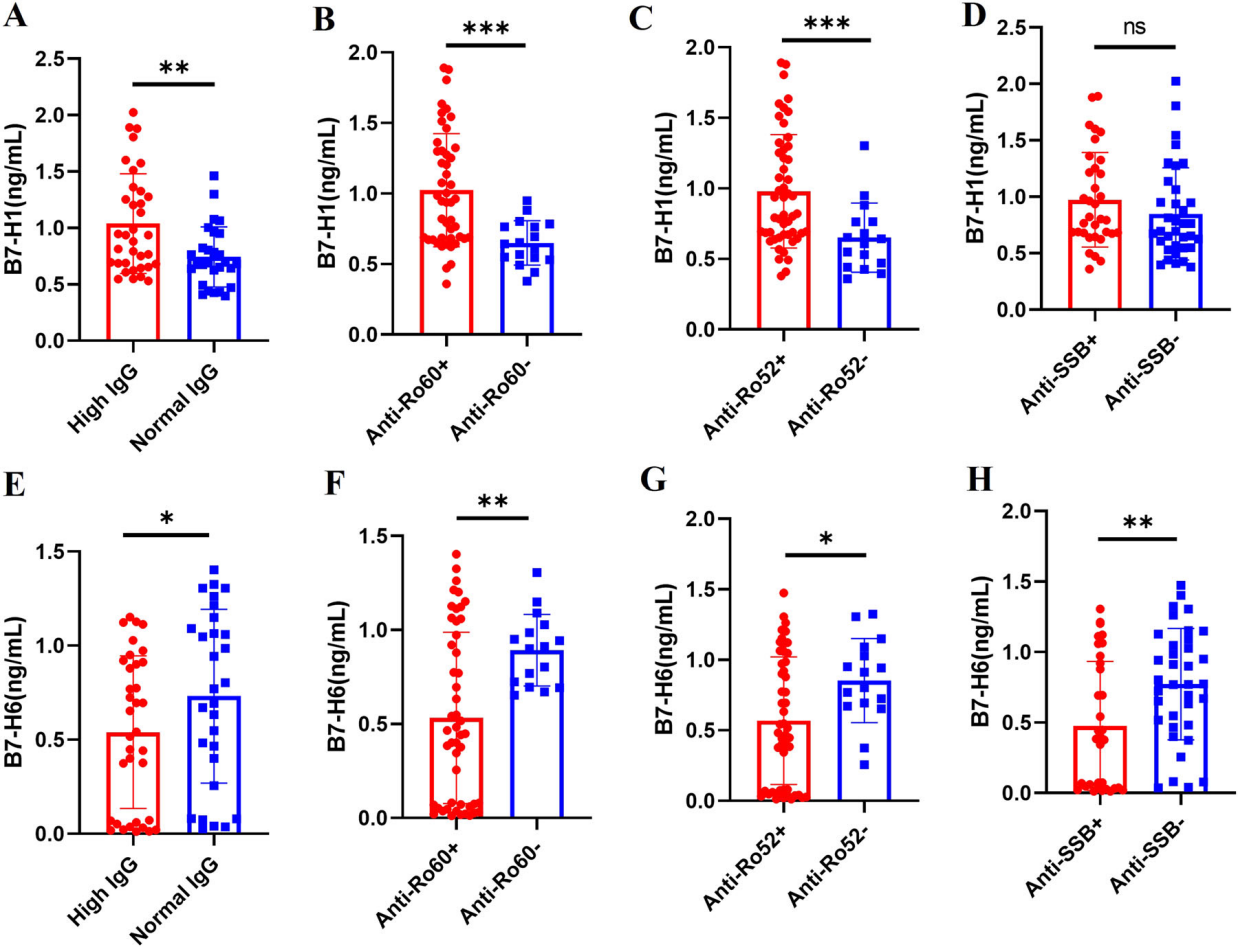
The expression levels of sB7‐H1 and sB7‐H6 in autoantibody‐positive and high IgG levels pSS patients. sB7‐H1 increased markedly in patients with high IgG (A, *p* = 0.008), anti‐Ro60 positive (B, *p* = 0.0002) and anti‐Ro52 positive (C, *p* = 0.001). sB7‐H1 levels were elevated in anti‐SSB positive patients (D, *p* = 0.13), but this increase was not statistically significant. sB7‐H6 decreased markedly in patients with high IgG (E, *p* = 0.04), anti‐Ro60 positive (F, *p* = 0.006), anti‐Ro52 positive (G, *p* = 0.03) and anti‐SSB positive (H, *p* = 0.004). Data of Figure 4 A, B, C, D, E and F used the Mann–Whitney *U*‐test for statistical difference analysis, *p < 0.05; **p < 0.01; ***p < 0.001; ns: not significant (*p* > 0.05).

We observed that sB7‐H1 expression was elevated in patients with high IgG (IgG ≥ 16 g/L) levels (0.94(0.68, 1.33) vs. 0.69(0.53, 0.92), *p *= 0.008) (Figure 4A), and those positive for anti‐Ro60 (0.95(0.68, 1.30) vs. 0.64(0.54(0.77), *p *= 0.0002) (Figure 4B) and anti‐Ro52 (0.82(0.67, 1.28) vs. 0.62(0.45, 0.76), *p *= 0.001) (Figure 4C) antibodies. While sB7‐H1 expression was also higher in anti‐SSB positive (0.81(0.68, 1.27) vs. 0.69(0.55, 1.06), *p *= 0.13) (Figure 4D) patients, the difference was not statistically significant. In contrast, sB7‐H6 expression was reduced in patients with high IgG levels (0.54(0.06, 0.91) vs. 0.77(0.33, 1.12), *p *= 0.04) (Figure 4E) and in those positive for anti‐Ro60(0.45(0.07, 1.01) vs. 0.91(0.70, 1.01), *p *= 0.006) (Figure 4F), anti‐Ro52(0.50(0.07, 1.00) vs. (0.86(0.68, 1.08), *p *= 0.03) (Figure 4G), and anti‐SSB (0.38(0.04, 0.95) vs. 0.77(0.49, 1.08), *p *= 0.004) (Figure 4H) antibodies.

### 3.7 ROC Curves Assess the Predictive Value of sB7‐H1 and sB7‐H6 for pSS Occurrence and Disease Activity

**Figure 5:**
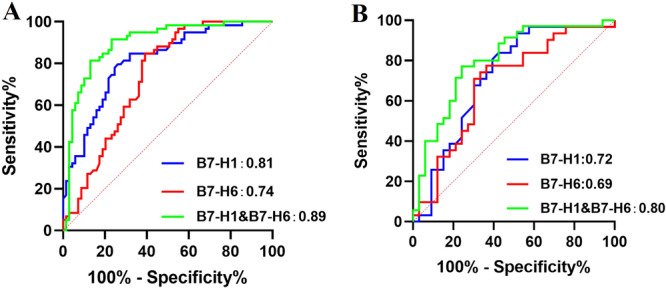
ROC curves assess the predictive value of sB7‐H1 and sB7‐H6 for pSS occurrence and disease activity. (A) The ROC curves for sB7‐H1, sB7‐H6, and their combined analysis show the effectiveness in distinguishing pSS, with AUC values of 0.81 (*p* < 0.0001), 0.74 (*p* < 0.0001), and 0.89 (*p* < 0.0001), respectively. (B) The ROC curves for sB7‐H1, sB7‐H6, and their combined analysis for distinguishing pSS disease activity, with AUC values of 0.72 (*p* = 0.003), 0.69 (*p* = 0.01), and 0.80 (*p* < 0.0001), respectively.

Our research findings revealed correlations between the expression levels of sB7‐H1 and sB7‐H6 in pSS patients and their clinical manifestations, laboratory parameters, and disease activity. As a result, we created receiver operating characteristic (ROC) curves to assess their diagnostic efficacy in predicting the onset (Figure 5A, Supporting Information S2: Table S1) and disease activity (Figure 5B, Supporting Information S2: Table S2) of pSS. The ROC curve for sB7‐H1 in distinguishing pSS showed an AUC of 0.81 (*p* < 0.0001), with 74% sensitivity and 80% specificity. sB7‐H6 had an AUC of 0.74 (*p* < 0.0001), with 72% sensitivity and 62% specificity. Their combined analysis resulted in an AUC of 0.89 (*p* < 0.0001), with 77% sensitivity and 88% specificity in distinguishing pSS. The ROC curve for sB7‐H1 in distinguishing pSS disease activity showed an AUC of 0.72 (*p* = 0.003), with 67% sensitivity and 74% specificity. sB7‐H6 had an AUC of 0.69 (*p* = 0.01), with 64% sensitivity and 69% specificity. Their combined analysis yielded an AUC of 0.80 (*p* < 0.0001), with 75% sensitivity and 74% specificity in distinguishing pSS disease activity.

## 4 Discussion

Aberrant immune responses in pSS are characterized by extensive infiltration of exocrine glands by circulating T and B lymphocytes, accompanied by the generation of autoantibodies such as anti‐SSA and anti‐SSB [15]. Immunotherapeutic approaches targeting co‐stimulatory molecules have shown promising advancements. CD40, a key costimulatory receptor essential for germinal center (GC) formation and function, has been closely associated with the pathogenesis of pSS. CFZ533, a novel monoclonal antibody that selectively blocks CD40 signaling, represents a potential therapeutic strategy aimed at modulating aberrant immune responses in pSS [16]. B7 co‐stimulatory molecules, expressed on antigen‐presenting cells (APCs) including dendritic cells and monocytes, are essential regulators of T cell activation and cytokine secretion by T helper cells [17]. Over time, multiple soluble B7 family members have been identified in the circulation and various tissues. These molecules play important roles in the dysregulation of immune responses and the pathogenesis of autoimmune diseases, representing a key focus of ongoing investigative efforts [18].

B7‐H1 (PD‐L1) has been extensively investigated in autoimmune diseases [19]. PD‐1 and its ligand PD‐L1 have been reported to play pivotal roles in mediating T cell co‐inhibition and promoting T cell exhaustion [20]. Soluble PD‐1 and PD‐L1 forms can amplify lymphocyte activity by inhibiting the PD‐1/PD‐L1 signaling pathway [21]. We speculate that this may be a potential reason for the elevated levels of sB7‐H1 observed in pSS patients. Sirui Qian et al. found that serum levels of PD‐L1 were significantly higher in patients with pSS compared to non‐pSS controls and HCs [22], which aligns with our findings. However, we expanded on this by analyzing the linear relationship between sB7‐H1 and both RF and CRP, as well as investigating differences in sB7‐H1 expression among patients with varying clinical characteristics and autoantibody levels. Furthermore, we employed ROC curve analysis to evaluate sB7‐H1's ability to differentiate pSS patients and assess disease activity.

B7‐H2 (ICOSL) is another molecule implicated in the regulation of T cell differentiation and activation [23]. While B7‐H2 was found elevated in pSS patients, particularly showing a positive correlation with IgG and ESR levels, further research is required to clarify its role in disease progression. The interaction between ICOSL and its receptor ICOS on T cells is known to regulate various CD4+ T cell subsets, including Th1, Th2, and Th17 cells [24], which are involved in the immune pathology of pSS. Our findings align with existing studies that have shown elevated B7‐H2 expression in pSS [17], suggesting a potential role in promoting the inflammatory response.

Regarding B7‐H3 (CD276) [25], its dual role as both a co‐stimulatory and co‐inhibitory molecule in autoimmune diseases [8] adds complexity to understanding its function in pSS. While previous studies have shown elevated B7‐H3 in the peripheral blood and salivary glands of pSS patients [26], our results did not find significant differences between pSS patients and HCs. However, we observed a negative correlation between B7‐H3 and RF, and a positive correlation with CRP, suggesting a potential modulatory role in inflammation, although further investigation with larger cohorts is necessary to confirm this hypothesis.

B7‐H4 has been identified as an inhibitor of immune responses in various in vitro studies [27]. Yu et al. reported significantly lower serum levels of sB7‐H4 in patients with pSS compared to HCs [28], which aligns with the trend observed in our study. However, the absence of statistical significance in our findings necessitates cautious interpretation. The exact role of B7‐H4 in pSS remains ambiguous and may be affected by the complex and dynamic nature of immune regulation in chronic autoimmune conditions. Further investigations are essential to elucidate the functional implications of B7‐H4 in this context.

Previous studies have found that B7‐H5 plays a significant role in various physiological and pathological processes, including the regulation of peripheral tolerance and the induction of T‐cell activation and differentiation [29]. Studies related to breast cancer have shown that sB7‐H5 is strongly correlated with CD8+ immune cell infiltration [30]. Currently, research on sB7‐H5 in autoimmune diseases is limited. Our findings revealed that sB7‐H5 expression was elevated in pSS patients compared to HCs and showed a positive correlation with RF, IgG, and ESR levels. However, more research is needed to understand the functional consequences of B7‐H5 expression in autoimmune settings.

B7‐H6, a ligand for the NK cell‐activating receptor NKp30 [31], has been studied in the context of cancer [32], but its role in autoimmune diseases remains poorly defined. Our study found lower levels of B7‐H6 in pSS patients compared to HCs, with a negative correlation with ESSDAI scores and clinical laboratory parameters. Given that NK cells are reduced in both number and activity in pSS patients [33], it is plausible that decreased B7‐H6 expression reflects impaired NK cell function, potentially contributing to the chronic immune dysregulation seen in pSS. However, this is speculative, and further research is required to confirm these findings.

In summary, while our study reveals novel associations between soluble B7 family molecules (sB7‐H1 and sB7‐H6) and the pathogenesis of pSS, several limitations should be acknowledged. First, the relatively small sample size may reduce the statistical power to detect subtle correlations between biomarker levels and clinical manifestations. Second, although the combined biomarker panel demonstrated promising diagnostic performance, its specificity for distinguishing pSS from closely related conditions—such as non‐Sjögren's sicca syndromes, SLE, and RA—remains unestablished. This represents a critical limitation, given the frequent serological overlap observed in clinical settings. Third, the single‐center design may constrain the generalizability of our findings to broader patient populations and different geographic regions. Additionally, the cross‐sectional nature of the study precludes longitudinal analysis of biomarker dynamics, limiting insights into their utility for monitoring therapeutic response or disease progression. Furthermore, we did not perform stratification based on individual ESSDAI domains, which may have provided more nuanced insights into the clinical associations of sB7‐H1 and sB7‐H6. However, the limited number of patients presenting with organ‐specific involvement—particularly in the pulmonary, renal, and central nervous system domains—precluded robust subgroup analyses. To address these limitations, future studies are planned to: (1) correlate circulating sB7‐H1 and sB7‐H6 levels with histopathological features—specifically, focal lymphocytic infiltration in salivary gland biopsies—to elucidate their tissue‐specific origins; (2) perform multicenter validation in ethnically and geographically diverse populations to confirm diagnostic reproducibility; (3) include cohorts of patients with other autoimmune diseases, such as RA and SLE, to evaluate the discriminatory potential of sB7‐H1 and sB7‐H6 expression—both individually and in combination—to more accurately determine their diagnostic value and specificity in distinguishing pSS from other clinically overlapping autoimmune disorders; (4) conduct prospective, longitudinal analyses to evaluate biomarker fluctuations in relation to immunosuppressive treatment response and disease trajectory; and (5) recruit larger and more heterogeneous patient cohorts to allow for stratified analyses based on individual ESSDAI domains. Particular attention will be paid to domains with limited representation in the current study—such as pulmonary, renal, and central nervous system involvement—to further explore the domain‐specific clinical relevance of sB7‐H1 and sB7‐H6. Moreover, ongoing sample collection, in combination with the systematic acquisition of ESSPRI and SSDI data, will be prioritized to enhance the ability to distinguish between healthy individuals and pSS patients, and to more comprehensively reflect the multifaceted nature of pSS through both clinical assessments and patient‐reported outcomes.

## 5 Conclusion

In summary, our study revealed that plasma sB7‐H1 and sB7‐H6 may serve as valuable biomarkers for identifying pSS patients and assessing the disease activity and severity of pSS. Additionally, to further validate their effectiveness as markers for evaluating disease activity in pSS, large‐scale studies are warranted to elucidate the functional roles of these molecules in the future.

## Author Contributions

**Saizhe Song:** writing – original draft, methodology, investigation, data curation. **Yanhong Yang:** validation, visualization, methodology, investigation. **Yu Shen:** data curation, formal analysis, methodology. **Tian Ren:** resources, methodology, investigation. **Zhiyong Sun:** funding acquisition, supervision. **Cuiping Liu:** funding acquisition, conceptualization, writing – review and editing, project administration.

## Conflicts of Interest

The authors declare no conflicts of interest.

## Supporting information


**Supplement Figure 1:** The expression levels of sB7‐H1 and sB7‐H6 across different clinical features and anti‐SSB positivity in pSS patients.

**Supplement Table 1:** Discriminative ability of B7‐H1, B7‐H6, and their combination in differentiating pSS patients from HCs. **Supplement Table 2:** Discriminative power of B7‐H1, B7‐H6, and their combination in assessing pSS disease activity.

## Data Availability

The data sets generated during and/or analysed during the current study are available from the corresponding author on reasonable request.
