# Supplementary figures and images for: Identification of Plasma Biomarkers for B7 Family Members Associated With Primary Sjögren's Syndrome
Source: Immun Inflamm Dis. 2025 Aug 22;13(8):e70250. doi: 10.1002/iid3.70250 (PMC12371553; doi:10.1002/iid3.70250)

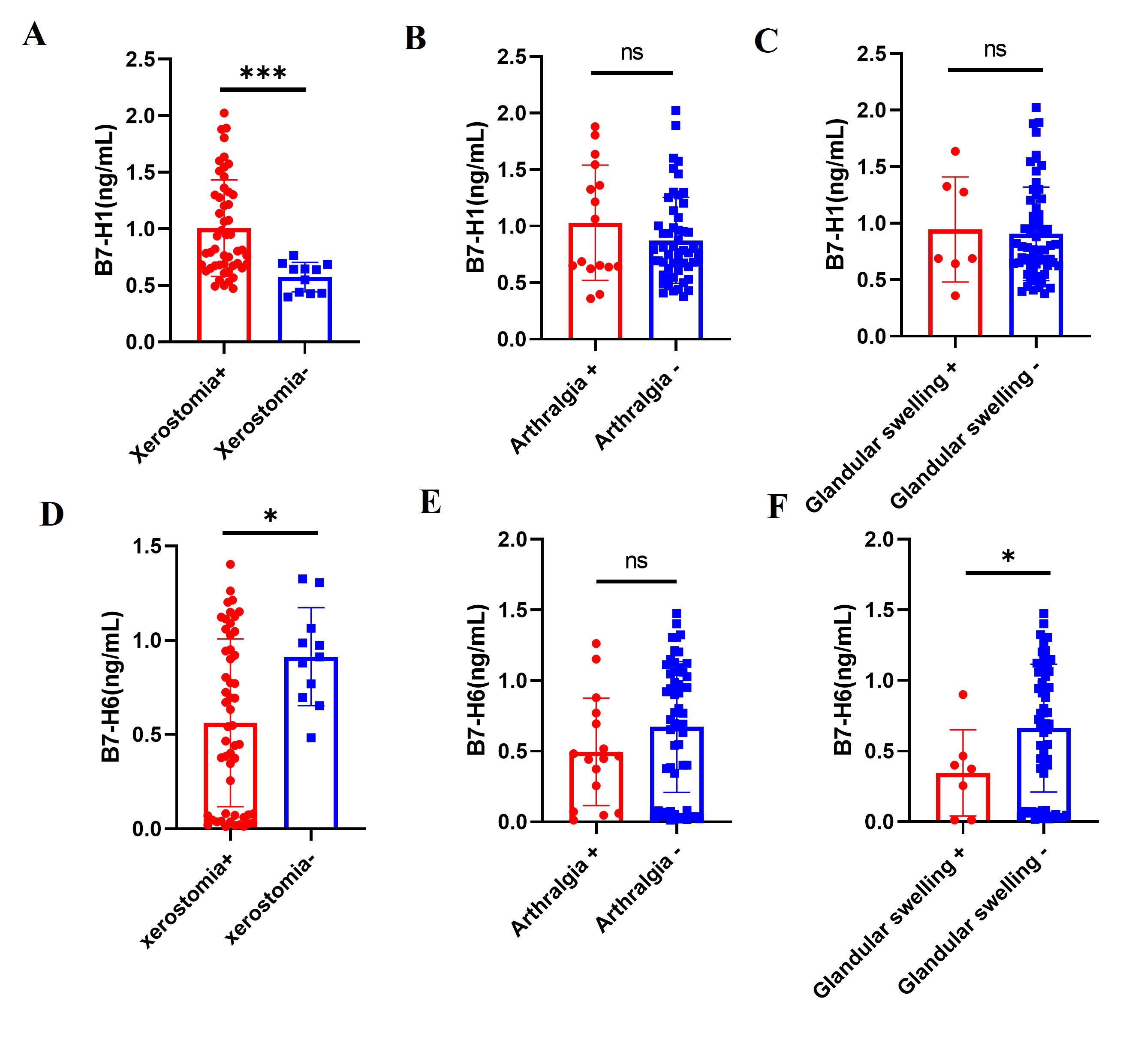

Supplement: Supplementary file 1 — Supplement Figure 1: The expression levels of sB7‐H1 and sB7‐H6 across different clinical features and anti‐SSB positivity in pSS patients. [file IID3-13-e70250-s001.jpg]
