# Supplementary material for: Identification of Plasma Biomarkers for B7 Family Members Associated With Primary Sjögren's Syndrome
Source: Immun Inflamm Dis. 2025 Aug 22;13(8):e70250. doi: 10.1002/iid3.70250 (PMC12371553; doi:10.1002/iid3.70250)
Supplement: Supplementary file 2 — Supplement Table 1: Discriminative ability of B7‐H1, B7‐H6, and their combination in differentiating pSS patients from HCs. Supplement Table 2: Discriminative power of B7‐H1, B7‐H6, and their combination in assessing pSS disease activity. [file IID3-13-e70250-s002.docx]

**Supplementary Material**

**Supplement Figure 1. The expression levels of sB7-H1 and sB7-H6 across different clinical features and anti-SSB positivity in pSS patients.** sB7-H1 increased in patients with xerostomia (A, *P* = 0.0002). sB7-H1 levels were elevated in patients with arthralgia (B, *P* = 0.51), glandular swelling (C, *P* = 0.50), however, these increases were not statistically significant. sB7-H6 decreased in patients with xerostomia (D, *P* = 0.02), glandular swelling (F, *P* = 0.04). sB7-H6 levels decreased in patients with arthralgia (E, *P* = 0.19), but this decrease was not statistically significant. Data of Figure A, B, C, D, E and F used the Mann – Whitney U - test for statistical difference analysis.

**Supplement Table 1** Discriminative ability of B7-H1, B7-H6, and their combination in differentiating pSS patients from HCs

| Characteristic | AUC | Cutoff value | Sensitivity (%) | Specificity (%) | *P* value | 95%CI |
| --- | --- | --- | --- | --- | --- | --- |
| B7-H1 | 0.81 | 0.64 | 74 | 80 | <0.0001 | 0.74~0.89 |
| B7-H6 | 0.74 | 0.78 | 72 | 62 | <0.0001 | 0.64~0.85 |
| B7-H1&B7-H6 | 0.89 | 0.62 | 77 | 88 | <0.0001 | 0.84~0.95 |

**Supplement Table 2** Discriminative power of B7-H1, B7-H6, and their combination in assessing pSS disease activity

| Characteristic | AUC | Cutoff value | Sensitivity (%) | Specificity (%) | *P* value | 95%CI |
| --- | --- | --- | --- | --- | --- | --- |
| B7-H1 | 0.72 | 1.07 | 67 | 74 | 0.003 | 0.59~0.85 |
| B7-H6 | 0.69 | 0.50 | 64 | 69 | 0.01 | 0.53~0.83 |
| B7-H1&B7-H6 | 0.80 | 0.47 | 75 | 74 | <0.0001 | 0.67~0.90 |
